# Inducible Nitric Oxide Synthase in Heart Tissue and Nitric Oxide in Serum of *Trypanosoma cruzi*-Infected Rhesus Monkeys: Association with Heart Injury

**DOI:** 10.1371/journal.pntd.0001644

**Published:** 2012-05-08

**Authors:** Cristiano Marcelo Espinola Carvalho, Jaline Coutinho Silverio, Andrea Alice da Silva, Isabela Resende Pereira, Janice Mery Chicarino Coelho, Constança Carvalho Britto, Otacílio Cruz Moreira, Renato Sergio Marchevsky, Sergio Salles Xavier, Ricardo Tostes Gazzinelli, Maria da Glória Bonecini-Almeida, Joseli Lannes-Vieira

**Affiliations:** 1 Laboratório de Biologia das Interações, Instituto Oswaldo Cruz (IOC)/Fiocruz, Rio de Janeiro, Rio de Janeiro, Brazil; 2 Serviço de Imunologia, Instituto de Pesquisa Clínica Evandro Chagas (IPEC)/Fiocruz, Rio de Janeiro, Rio de Janeiro, Brazil; 3 Departamento de Patologia, Universidade Federal Fluminense, Niterói, Rio de Janeiro, Brazil; 4 Serviço de Anatomia Patológica, IPEC/Fiocruz, Rio de Janeiro, Rio de Janeiro, Brazil; 5 Laboratório de Biologia Molecular e Doenças Endêmicas, IOC/Fiocruz, Rio de Janeiro, Rio de Janeiro, Brazil; 6 Laboratório de Neurovirulência, BioManguinhos/Fiocruz, Rio de Janeiro, Rio de Janeiro, Brazil; 7 Laboratório de Imunoparasitologia, Instituto Rene Rachou/Fiocruz, Belo Horizonte, Minas Gerais, Brazil; 8 Departamento de Imunologia e Bioquímica, Instituto de Ciências Biológicas (ICB), Universidade Federal de Minas Gerais, Belo Horizonte, Minas Gerais, Brazil; René Rachou Research Center, Brazil

## Abstract

**Background:**

The factors contributing to chronic Chagas' heart disease remain unknown. High nitric oxide (NO) levels have been shown to be associated with cardiomyopathy severity in patients. Further, NO produced via inducible nitric oxide synthase (iNOS/NOS2) is proposed to play a role in *Trypanosoma cruzi* control. However, the participation of iNOS/NOS2 and NO in *T. cruzi* control and heart injury has been questioned. Here, using chronically infected rhesus monkeys and iNOS/NOS2-deficient (*Nos2* ^−/−^) mice we explored the participation of iNOS/NOS2-derived NO in heart injury in *T. cruzi* infection.

**Methodology:**

Rhesus monkeys and C57BL/6 and *Nos2* ^−/−^ mice were infected with the Colombian *T. cruzi* strain. Parasite DNA was detected by polymerase chain reaction, *T. cruzi* antigens and iNOS/NOS2^+^ cells were immunohistochemically detected in heart sections and NO levels in serum were determined by Griess reagent. Heart injury was assessed by electrocardiogram (ECG), echocardiogram (ECHO), creatine kinase heart isoenzyme (CK-MB) activity levels in serum and connexin 43 (Cx43) expression in the cardiac tissue.

**Results:**

Chronically infected monkeys presented conduction abnormalities, cardiac inflammation and fibrosis, which resembled the spectrum of human chronic chagasic cardiomyopathy (CCC). Importantly, chronic myocarditis was associated with parasite persistence. Moreover, Cx43 loss and increased CK-MB activity levels were primarily correlated with iNOS/NOS2^+^ cells infiltrating the cardiac tissue and NO levels in serum. Studies in *Nos2* ^−/−^ mice reinforced that the iNOS/NOS2-NO pathway plays a pivotal role in *T. cruzi*-elicited cardiomyocyte injury and in conduction abnormalities that were associated with Cx43 loss in the cardiac tissue.

**Conclusion:**

*T. cruzi*-infected rhesus monkeys reproduce features of CCC. Moreover, our data support that in *T. cruzi* infection persistent parasite-triggered iNOS/NOS2 in the cardiac tissue and NO overproduction might contribute to CCC severity, mainly disturbing of the molecular pathway involved in electrical synchrony. These findings open a new avenue for therapeutic tools in Chagas' heart disease.

## Author Summary

Chagas disease, a neglected tropical disease caused by the protozoan *Trypanosoma cruzi*, afflicts from 8 to 15 million people in the Latin America. Chronic chagasic cardiomyopathy (CCC) is the most frequent manifestation of Chagas disease. Currently, patient management only mitigates CCC symptoms. The pathogenic factors leading to CCC remain unknown; therefore their comprehension may contribute to develop more efficient therapies. In patients, high nitric oxide (NO) levels have been associated with CCC severity. In *T. cruzi*-infected mice, NO, mainly produced via inducible nitric oxide synthase (iNOS/NOS2), is proposed to work in parasite control. However, the participation of iNOS/NOS2 and NO in *T. cruzi* control and heart injury has been questioned. Here, infected rhesus monkeys and iNOS/NOS2-deficient mice were used to explore the participation of iNOS/NOS2-derived NO in heart injury in *T. cruzi* infection. Chronically infected monkeys presented electrical abnormalities, myocarditis and fibrosis, resembling the spectrum of human CCC. Moreover, cardiomyocyte lesion correlated with iNOS/NOS2^+^ cells infiltrating the cardiac tissue. Our findings support that parasite-driven iNOS/NOS2^+^ cells accumulation in the cardiac tissue and NO overproduction contribute to cardiomyopathy severity, mainly disturbing the pathway involved in electrical synchrony in *T. cruzi* infection.

## Introduction

Chagas disease, which is caused by the protozoan parasite *Trypanosoma cruzi*, afflicts 8–15 million individuals in endemic areas of Latin America and several hundred thousand people in other countries as a result of migration. Although vector transmission has been controlled, there are still more than 50,000 new cases of Chagas disease each year [1], [2]. Despite high parasitism, which usually declines at immunity onset, the clinical signs are usually mild in the acute infection. After decades, most of the infected individuals remain in the asymptomatic indeterminate form, and ∼30% of the patients present arrhythmias and heart failure due to end-stage dilated chronic chagasic cardiomyopathy (CCC), which is associated with inflammation, myocytosis and fibrosis [2]. The pathophysiological factors influencing the clinical outcome of Chagas disease remain unclear [2]. Due to the scarcity of the *T. cruzi* parasite, CCC has been associated with autoimmune recognition of heart tissue by T-cell-enriched inflammation [3]. There is a consensus, however, that parasite persistence and/or a parasite-driven deregulated immune response operates in CCC [4], [5]. In this context, high nitric oxide (NO) levels have been shown to be associated with the severity of CCC in chronic chagasic patients [6]. Nitric oxide is an important cytotoxic and cytostatic factor in cell-mediated immunity to intracellular pathogens [7]. Excessive NO, however, may cause host injury, including a reduction of myocardial contractibility [8]. Nitric oxide is formed from L-arginine by isoforms of NO synthase (NOS): the constitutive isoforms, neuronal NOS (nNOS/NOS1) and endothelial NOS (eNOS/NOS3), and the cytokine-inducible NOS (iNOS/NOS2) [8]. In *T. cruzi* infection, *in vitro* and *in vivo* evidence support that NO plays a pivotal role as a first line of parasite growth control [9]. Nevertheless, iNOS/NOS2-derived NO takes part in ventricular dilation and systolic dysfunction in *T. cruzi*-elicited acute myocarditis [10]. In chagasic patients, high NO levels have been shown to be associated with the severity of CCC [6]. Further, iNOS/NOS2 has been shown to contribute to *T. cruzi* control in acute infection [10], [11]. This role, however, was challenged in a study with iNOS/NOS2-defcient (Nos2^tm1Lau^) infected mice showing that iNOS/NOS2 is not required for control of *T. cruzi* growth [12]. Moreover, iNOS/NOS2 participation in pathology has been questioned by study of gene polymorphism at promoter region in patients [13]. Therefore, there are doubts about the role played by iNOS/NOS2 and NO in *T. cruzi* infection. Adopting the model of nonhuman primate rhesus monkeys (*Macaca mulatta*) chronically infected with *T. cruzi* that reproduced several clinical, parasitological and immunological features of Chagas disease [14], we descriptively investigated the involvement of iNOS/NOS2 and NO in Chagas' heart disease. Further, promptly, iNOS/NOS2-deficient mice were used to add insights on the participation of iNOS/NOS2-derived NO in *T. cruzi*-elicited heart injury.

## Materials and Methods

### Ethics statement

This study was carried out in strict accordance with the recommendations in the Guide for the Care and Use of Laboratory Animals of the Brazilian National Council of Animal Experimentation (http://www.cobea.org.br/) and the Federal Law 11.794 (October 8, 2008). The Institutional Committee for Animal Ethics of Fiocruz (CEUA/Fiocruz, Licenses 161/03 and 004/09) and the Biosafety National Committee (CQB/CTNBio, License 105/99) approved all the procedures used in this study.

### Animals and infection

Seven male rhesus monkeys (*Macaca mulatta*, 26±1.7 years old) chronically infected with *T. cruzi* (monkeys #42, #64, #68, #90, #95, #99, #103) were individually caged in the nonhuman primate units (Double L Group Ltd., USA) of the Nonhuman Primates Breeding Service (SCPRIM), of the Laboratory of Animals Breeding Center at Fiocruz (CECAL/Fiocruz, Rio de Janeiro, Brazil). The monkeys were provided with water *ad libitum* and fed a commercial chow (Nuvilab Primates 6030, Nuvital, Brazil) that was supplemented daily with fruits, eggs and vegetables. Temperature, humidity and light/dark cycles were standardized. Two noninfected age-matched male monkeys (#81 and #94) were analyzed as controls. Four noninfected male monkeys (L17, L21, M31, N31) from our colony were used as controls for heart function evaluations.

Metacyclic trypomastigotes of the Colombian *T. cruzi* strain were used to infect the monkeys subcutaneously in the arm [15]. These animals were studied during the acute and early chronic infection [15]. In this follow up study, the chronically infected monkeys were analyzed at 16–19 years postinfection (ypi) and followed for 48 months. The animals did not receive etiological treatment for *T. cruzi*. Examinations and procedures were performed under anesthesia with 10 mg/Kg ketamine chloride (Vetaset, Fort Dodge, Iowa, USA) intramuscularly according to the *Guide for the Care and Use of Laboratory Animals* (NIH Publication No. 85-23, revised 1996). Blood was obtained by puncture of the femoral vein with appropriate tubes (Vacutainer, Becton & Dickinson, USA). Prior to necropsy, monkeys were sedated with ketamine chloride and euthanized by exsanguination under deep plane of sodium thiopental (Thiopentax, Cristalia, Belo Horizonte, MG, Brazil). Samples of the heart and all major organs were taken for histological studies and polymerase chain reaction (PCR) for parasite kDNA and genomic DNA detection. For the histopathology study, heart samples taken from five infected monkeys, which were sacrificed at the parasitemia peak (41 days postinfection (dpi), monkeys #37 and #67), when parasitemia was negative (70 dpi, monkey #77, and 76 dpi, monkey #93) and 3 years postinfection (monkey #45) used in a previous work [15] were added to our study.

Five- to seven-week-old female C57BL/6 (H-2^b^) and iNOS-deficient (*Nos2* ^−/−^; B6.129P2-Nos2^tm1Lau^/J) mice, resulted of seven backcrossings of the original *Nos2*-deficient lineage in C57BL/6 mice, were obtained from the animal facilities of Fiocruz and were maintained in specific pathogen free conditions. The mice were infected intraperitoneally with 100 blood trypomastigotes of the Colombian strain [16].

### Electrocardiogram, echocardiogram and radiology

The classic 12-lead human electrocardiogram (ECG) system was used to analyze rhesus monkeys. Tracings were made at 25 mm/s at a voltage of 1 mV standardized to 1 cm (ECG-6, Ecafix, Brazil) [14]. Two-dimensional and M-mode echocardiogram (ECHO) was performed on a regular basis and recorded with a multi-image camera (Ultrasound Scanner EUB-555, Hitachi, Japan). The ventricular function was assessed in the M-mode, by calculating the ejection fraction, and in the bidimensional mode, by semiquantitatively analyzing the global systolic function [14]. Electrocardiogram, ECHO and radiology (chest, esophagus, colon) were performed at 12-month intervals.

Mice were intraperitoneally tranquilized with diazepam (20 mg/Kg), and transducers were placed under the skin for DII derivation. Electrocardiogram traces were recorded during two minutes using Power Lab 2/20 (PanLab Instruments, Spain), analyzed with Scope Software for Windows v3.6.10 (PanLab Instruments, USA) [16] and independently analyzed by two investigators.

### Histopathology studies

Tissue fragments were fixed in 10% formalin, processed, and embedded in paraffin. Tissue sections (5 µm) were stained with hematoxylin and eosin (H&E) and immunohistochemically (IHS) stained. To evaluate collagen deposits, heart sections were stained with Siriusrot F3B (Chroma Gessellschaft, Germany) in a saturated aqueous solution of picric acid and fast green. The proportion of collagen-positive areas was evaluated with the digital morphometric apparatus and analyzed with AnalySIS AUTO Software (Soft Imaging System, USA).

### Immunohistochemical staining

A polyclonal anti-iNOS/NO2 antibody was obtained from Cayman Chemical (#160862, USA), and an anti-connexin 43α1 (Cx43) polyclonal antibody was purchased from Sigma (#C6219, USA). In addition, a polyclonal antibody recognizing *T. cruzi* antigens was produced in rabbits (LBI/IOC-Fiocruz, Brazil). A biotinylated anti-rabbit and peroxidase-streptavidin complex was purchased from Amersham (England). Antibodies and reagents were utilized in compliance with the manufacturers' instructions. Serial 5-µm sections were subjected to standardized IHS [16]. The material was counterstained with Mayer's hematoxylin. The *T. cruzi-*, iNOS/NOS2- or Cx43-positive areas in 25 fields (12.5 mm^2^) per section, in 3 sections per heart, were evaluated with a digital morphometric apparatus. For the quantitative studies of Cx43 expression only areas with preserved myocardial cells were analyzed. The images were analyzed with AnalySIS AUTO Software (Soft Imaging System, USA). The areas that expressed the molecule of interest were integrated with the areas that did not express the molecule of interest, and the data were presented as the percentage of the positive area.

### DNA extraction and PCR conditions

Two or three different tissue samples per studied organ were processed separately for DNA extraction, and the purified DNA was PCR amplified using *T. cruzi*-specific kDNA minicircle primers [14] or analyzed by real time quantitative PCR (qPCR) using primers directed to the nuclear satellite DNA [17]. DNA integrity and the possible presence of PCR inhibitors were checked by amplification of the human β-globin sequence [14]. Samples showing no amplification for β-globin were retested after a new DNA extraction. For the real time PCR assays heart and spleen samples (of one to two cm^3^) kept in liquid nitrogen were sectioned using a cryostat in conditions to avoid DNA cross-contamination and the studied area calculated using the AnalySIS AUTO Software (Soft Imaging System, USA). As negative and positive controls, respectively, heart tissues from noninfected C57BL/6 mice or mice at 50 dpi with the Colombian *T. cruzi* strain were used [16].

The qPCR experiments were performed using the ABI Prism 7500 Fast (Applied Biosystems, USA), in a final volume of 20 µL containing 2 µL DNA samples and 10 µl GoTaq qPCR Master Mix (Promega, USA). Primers Cruzi 1 and Cruzi 2 were used for the *T. cruzi* nuclear satellite target [17]. The cycling conditions were as follows: 95°C for 10 minutes, followed by 40 cycles at 95°C for 15 seconds and 58°C for 1 minute. After amplification, the specificity of these primers was confirmed through melting curve analysis of the generated amplicons, revealing a solely melting temperature (Tm) for the amplified fragment. Each sample submitted to qPCR analysis was performed in triplicates and the results were expressed as mean values. Parasitic load quantification was obtained by absolute quantification of *T. cruzi* DNA, following normalization of the heart and spleen analyzed areas. The standard curve was generated by a 1∶10 serial dilution of DNA extracted from *T. cruzi* Colombian epimastigotes culture stocks, ranging from 10^6^ to 10 parasite equivalents.

### Total IgG and IgM and specific anti-*T. cruzi* serology

The levels of total IgG and IgM were determined by kinetic nephelometry (Beckman Coulter Array 360, Beckman Coulter, USA) in accordance with the manufacturer's instructions. Specific anti-*T. cruzi* IgG antibodies were measured by ELISA (EIE-Chagas, BioManguinhos, Fiocruz, Brazil) in accordance with the manufacturer's instructions.

### Nitric oxide quantification

Nitrate and nitrite were determined in plasma samples from noninfected and infected monkeys using Griess reagent and vanadium chloride III with a standard curve of 0.8–100 µM NaNO_2_ and NaNO_3_ [18].

### Creatine kinase detection

The activity of the CK-MB isoenzyme was measured in serum with a commercial kit (Labtest, Brazil) according to the manufacturer's recommendations. The optical density at 340 nm (Microplate Reader Benchmark, Bio-Rad, USA) was recorded every 2 minutes for 15 minutes [16].

### Statistical analysis

Data are expressed as arithmetic mean ± SD. Student's *t* test was adopted to analyze the statistical significance of the apparent differences. All statistical tests were performed with SPSS 8.0 software. Differences were considered statistically significant at *p*<0.05.

## Results

### Spectrum of clinical forms of chronic Chagas disease is reproduced in *Trypanosoma cruzi*-infected rhesus monkeys

The general characteristics of the studied monkeys are shown in Table S1. Electrical conduction abnormalities found in acute and chronically *T. cruzi*-infected monkeys are summarized in Table 1. Interestingly, the mild electrical abnormalities that we detected during the acute *T. cruzi* infection disappeared at the 4^th^ month postinfection [15]. In this follow up study, during the chronic infection (18–23 ypi), three monkeys (#42, #64 and #68) showed transient electrical alterations, and three animals (#90, #95 and #103) presented significant electrical conduction abnormalities. The ECG patterns were normal in the 4 analyses of one monkey (#99). In infected monkey #90, multiform ventricular extrasystoles were observed at 16 ypi, atrial extrasystoles were observed at 17 ypi, an incomplete left bundle branch block (LBBB) was observed at 20 ypi, and T-wave inversion was observed in all 4 analyses. In monkey #103, first-degree atrioventricular (AV) conduction disturbance and T-wave inversion were seen at 17 ypi. In monkey #95, LBBB was seen in the 4 exams, and the T-wave inversion was more accentuated and right QRS axis deviation at 20 ypi. Representative ECG registers of chronically *T. cruzi*-infected are shown in Figure S1. As shown in Table S2, only monkey #95 showed ECHO abnormality with asynchronic interventricular septum motility and a decreased left ventricular ejection fraction (72.4% and 53.2% at 16 ypi and 17 ypi, respectively). Altogether, monkeys #42, #64, #68 and #99 were considered non-cardiopatic and monkeys #90, #95 and #103 were considered cardiopatic. All *T. cruzi*-infected monkeys showed normal radiological exams of the chest, esophagus and colon at the end-point of this study (data not shown). Therefore, monkeys #42, #64, #68 and #99 presented the indeterminate form of the chronic Chagas disease.

**Table 1:** Electrocardiographic patterns detected in *Trypanosoma cruzi*-infected rhesus monkeys during acute and chronic infection.

	Acute phase*		Chronic phase							
Monkeys	Weeks p.i.	Heart condition	Years p.i.	Heart condition	Years p.i.	Heart condition	Years p.i.	Heart condition	Years p.i.	Heart condition
42	8–12	AVB, RBBB†	19	AVR	20	Normal	20	Sacrificed	-	-
64	20	Normal	19	AVR	20	Normal	22	Normal	23	Normal
68	4–12	AVB, RBBB,LvQRS	18	Sacrificed	-	-	-	-	-	-
99	20	Normal	16	Normal	17	Normal	19	Normal	20	Normal
103	6–8	AVB, AVR	16	Normal	17	AVB, AVR	19	AVB	20	RBBB
90	4–8	AVB,LvQRS	16	Ventricular extrasystoles, AVR	17	Atrial extrasystoles, AVR	19	AVR	20	LBBB,AVR
95	4–12	AVB, AVR	16	LBBB	17	LBBB, AVR	19	LBBB	20	LBBB, AVR, RAD

*: [15].

**†:** ECG patterns were evaluated using the following standard criteria: AVB - atrioventricular block, AVR - Abnormal ventricular repolarization, LBBB - Left bundle branch block - second-degree His bundle, LvQRS - Low voltage QRS, RBBB - right bundle branch block - first-degree His bundle, RAD - right QRS axis deviation.

### *Trypanosoma cruzi*-infected rhesus monkeys presented histopathological alterations compatible with chronic chagasic cardiomyopathy

In the acute infection, intense heart parasitism was associated with pronounced inflammatory infiltrates at 41 dpi (monkeys #37 and #67). This effect subsided at 76dpi (monkey #93), which coincided with parasitemia control. Inflammation was focal or absent at 3 ypi (monkey #45) [15]. In the chronic infection, none of the analyzed organs showed macroscopic alterations. In addition, no ventricular conduction system alteration was observed in the analyzed areas of the fibers composing the bundle of His in chronically *T. cruzi*-infected rhesus monkeys (data not shown). The main histopathological alterations found in the cardiac tissue are represented in the Figure S2. In monkeys with electrical abnormalities (#90, #95 and #103) and decreased left ventricular ejection fraction (#95), histopathological alterations were heterogeneous with areas with normal aspect (Figure S2E), but mostly areas with a mild or intense multifocal myocarditis (more pronounced in the left ventricle), with lymphocytes, macrophages, and an apparent lack of parasites, was associated with the disrupted spaces between cardiomyocytes and hypertrophy of the myocardial fibers (Figure S2D, Figure S2F). Further, monkeys with the indeterminate form of Chagas disease presented focal myocarditis (Figure S2A, Figure S2B) or complete absence of cardiac inflammation or myocardial fiber injury (Figure S2C). Conversely, noninfected controls (Figure S2G) consistently did not have inflammation or myocardial fiber injury.

**Figure 1:**
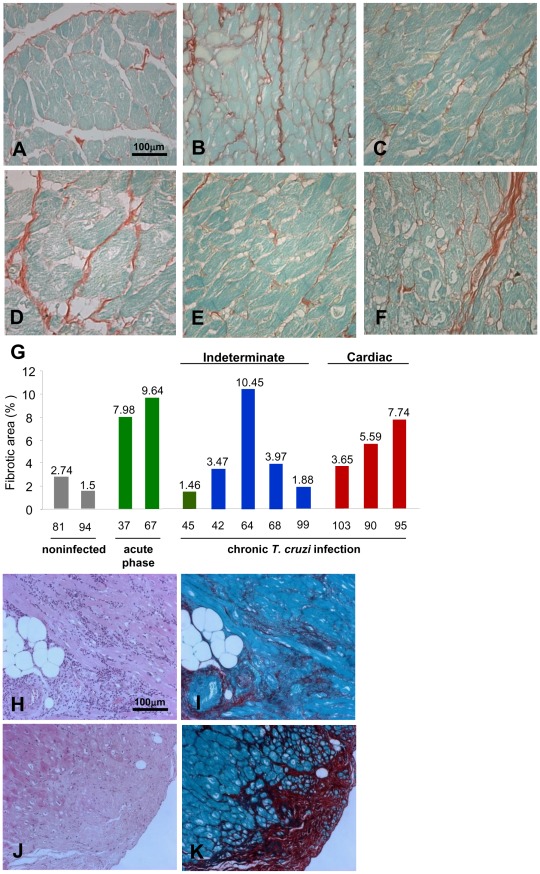
Increased collagen deposits in the myocardium of *T. cruzi*-infected rhesus monkeys. Collagen deposition was used to assess fibrosis in the myocardium of the left ventricle of noninfected and *T. cruzi*-infected monkeys. (**A**) Noninfected monkey #94, normal slight collagen deposits. (**B**) Monkey #67 (41 dpi), increased collagen deposition. (**C**) Monkey #45 (3 ypi), normal collagen deposits. (**D**) Monkey #64 (23 ypi), increased collagen deposition. (**E**) Monkey #99 (20 ypi) normal slight collagen deposits. (**F**) Monkey #95 (20 ypi), increased interstitial matrix deposits. **G.** Percentage of cardiac section area occupied by collagen deposits in the myocardium of the left ventricle of noninfected and *T. cruzi*-infected monkeys. (**H–K)** Serial heart sections of monkey #95 (20 ypi) showing: (**H**) intense infiltrates of mononuclear inflammatory cells (**I**) paralleling fibrosis, and (**J**) substitution of cardiomyocytes by mesenchymal cells in (**K**) an area of intense fibrosis. **A–F**, **I** and **K**, Picro-Sirius red stain. **H** and **J**, H&E. Bar = 100 µm.

Compared with noninfected controls (Figure 1A, Figure 1G), myocardial fibrosis with collagen deposits was detected in the early (41 dpi) acute infection (Figure 1B, Figure 1G). Fibrosis was residual in the early (3 ypi) chronic infection (Figure 1C, Figure 1G) and characterized the indeterminate form of the disease. In the late (20 ypi) chronic infection, the monkey that had transient alterations in clinical score (#64) presented intense fibrosis (Figure 1D, Figure 1G), while in the monkey with normal clinical score (#99) heart histological aspects and collagen deposits resembling those of noninfected controls (Figure 1E, Figure 1G). In the cardiopatic monkeys (#90, #95 and #103), fibrosis (Figure 1F, Figure 1G) was related to the severity of electrical abnormalities. Collagen deposits varying from slight to severe were frequently associated with inflammatory foci (Figure 1H–K). In areas of severe inflammation (Figure 1H), fibrosis was abundant (Figure 1I), and there was substitution of cardiomyocytes by mesenchymal cells (Figure 1J) and dense bundles of interstitial matrix that extended from the subepicardium into the subjacent myocardium (Figure 1K). Therefore, in six out of seven *T. cruzi*-infected rhesus monkeys, clinical and histological alterations were compatible with chronic Chagas disease. Interestingly, three of these monkeys reproduced major aspects of CCC in patients [2], [19].

### Parasite persistence in chronically *Trypanosoma cruzi*-infected monkeys

**Figure 2:**
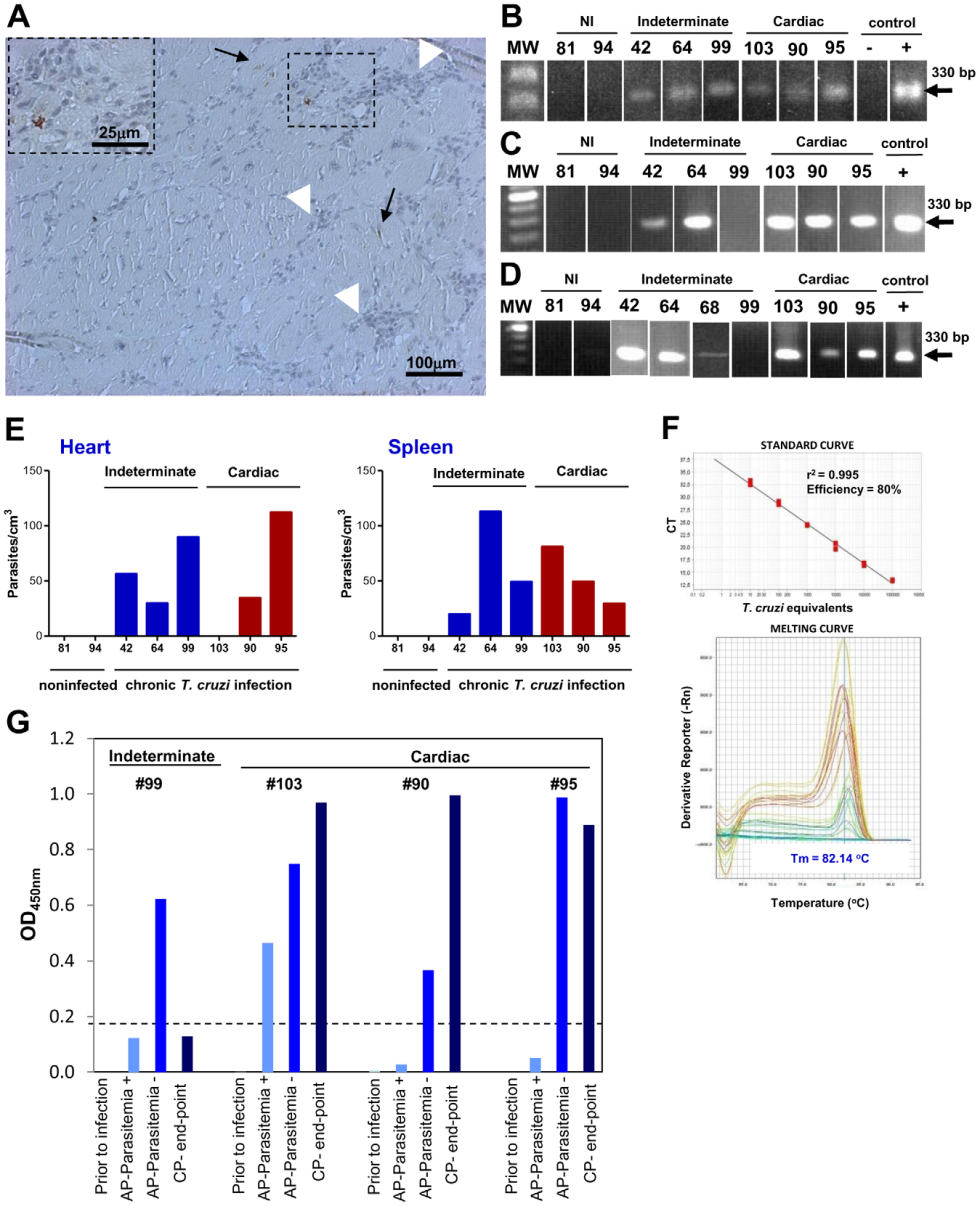
Persistence of *T. cruzi* in chronically infected rhesus monkeys. The persistence of *T. cruzi* parasite and antigens was evaluated by immunohistochemistry, PCR and antibody response. (**A**) Photomicrographs of section of myocardium of left ventricle of monkey #95 (20 ypi). Immunohistochemistry for *T. cruzi* antigens (black arrows and insert) associated (dotted square) or not associated (black arrow) with focal inflammation. Inflammatory infiltrates lacking parasite antigens (white arrow heads). **B–C**. PCR for *T. cruzi* kDNA (∼330 bp) in blood of noninfected controls (NI) and *T. cruzi*-infected rhesus monkeys at (**B**) 16–20 ypi and (**C**) 20–23 ypi. (**D**) PCR for *T. cruzi* kDNA (∼330 bp) in fragments of the left ventricle (LV) of the heart of noninfected controls and *T. cruzi*-infected rhesus monkeys at 20–23 ypi. Negative (−) and positive (+) controls were heart fragments of noninfected and *T. cruzi*-infected C57BL/6 mice, respectively. (**E**) Real time qPCR for the *T. cruzi* satellite DNA sequences Cruzi1/Cruzi2 in heart and spleen of noninfected controls and *T. cruzi*-infected rhesus monkeys at 20–23 ypi. (**F**) Standard curve of 10-fold serial dilution of DNA of epimastigote forms of the Colombian *T. cruzi* strain (10^6^ to 10 parasites/mL) used for the absolute quantification by real time qPCR. The linear regression curve, coefficient of determination (r^2^ = 0.995) and qPCR efficiency (E = 80%) are indicated. The melting curve is also shown. (**G**) Serology for IgG anti-*T. cruzi* in rhesus monkeys prior to infection, during the acute phase (AP), when parasitemia was positive (+) and negative (−), and during the chronic phase (CP; at the end-point 20 ypi). Bar = 100 µm; Bar = 25 µm in insert in (**A**).

Exhaustive microscopic examinations of paraffin heart tissue sections failed to detect *T. cruzi* pseudocysts or isolated amastigotes in all of the chronically infected monkeys. Immunohistochemistry experiments were performed to detect *T. cruzi* antigens, and extramyocytic antigens were seen as red amorphous spots (Figure 2A) in six out of seven infected animals (the exception was monkey #99). *T. cruzi* antigens were commonly surrounded by mononuclear cell infiltrates (Figure 2A, black arrows); however, inflammatory cells were also seen associated to apparently noninfected fibers (Figure 2A, white arrow heads). In addition, blood samples and fragments of spleen and cardiac tissue were submitted to conventional PCR amplification targeting *T. cruzi* kDNA minicircles [14]. In the 1^st^ analysis (16–19 ypi) of blood, all *T. cruzi*-infected animals showed a PCR signal at ∼330 bp for kDNA, whereas the noninfected monkeys (#81 and #94) did not show a signal (Figure 2B). In the 4^th^ analysis (20–23 ypi), a PCR signal at ∼330 bp was detected in the blood of six infected rhesus monkeys (Figure 2C). Furthermore, *T. cruzi* kDNA was identified in the cardiac septum, left (Figure 2D) and right ventricles, left and right atria, and aorta of all infected monkeys except monkey #99. All of the infected monkeys were parasitologically positive for *T. cruzi* at 16–19 ypi [14]. At 20 ypi, however, attempts to identify *T. cruzi* kDNA in the blood and heart of monkey #99 were consistently negative (Figure 2C, Figure 2D). Considering that low parasitism could contribute to these results, we performed additional studies using two or three tissue samples per analyzed monkey. Real time qPCR for the conserved repetitive nuclear satellite DNA sequences revealed low parasitism in heart samples of all *T. cruzi*-infected rhesus monkeys except monkey #103. Conversely, real time qPCR revealed low parasitism in spleen samples of all *T. cruzi*-infected monkeys (Figure 2E, Figure 2F). Heart and spleen samples of noninfected controls were repeatedly negative (Figure 2E). The β-globin sequence was detected as a control for DNA integrity and PCR inhibition in all analyzed samples (data not shown). Taken together, these results support parasite persistence in indeterminate (#42, #64, #68, #99) and cardiopatic (#90, #95, #103) chronically *T. cruzi*-infected rhesus monkeys. Because antibody detection is a criterion for persistent infection [20], we analyzed the kinetics of the anti-*T. cruzi* antibody in a group of infected monkeys that were cardiopatic (#90, #95 and #103) and a monkey that was indeterminate (#99) at 20 ypi. Figure 2G shows that all monkeys were seronegative prior to infection. The anti-*T. cruzi* antibody was only detected in one monkey (#103) when parasitemia was positive (18 dpi). When parasitemia was controlled (59 dpi) [15] all of the infected monkeys presented anti-*T. cruzi* antibodies. At the end-point of our study, anti-*T. cruzi* antibodies were detected in cardiopatic monkeys (#90, #95, #103), but not in indeterminate monkey #99. Importantly, there were no alterations in the levels of total IgM (Figure S3A) and IgG (Figure S3B) in serum of *T. cruzi*-infected monkeys in comparison with noninfected controls at the end-point of this study (20–23 ypi).

### iNOS/NOS2^+^ cells in the cardiac tissue and high NO levels in serum are associated with cardiomyopathy in chronically *Trypanosoma cruzi*-infected monkeys

**Figure 3:**
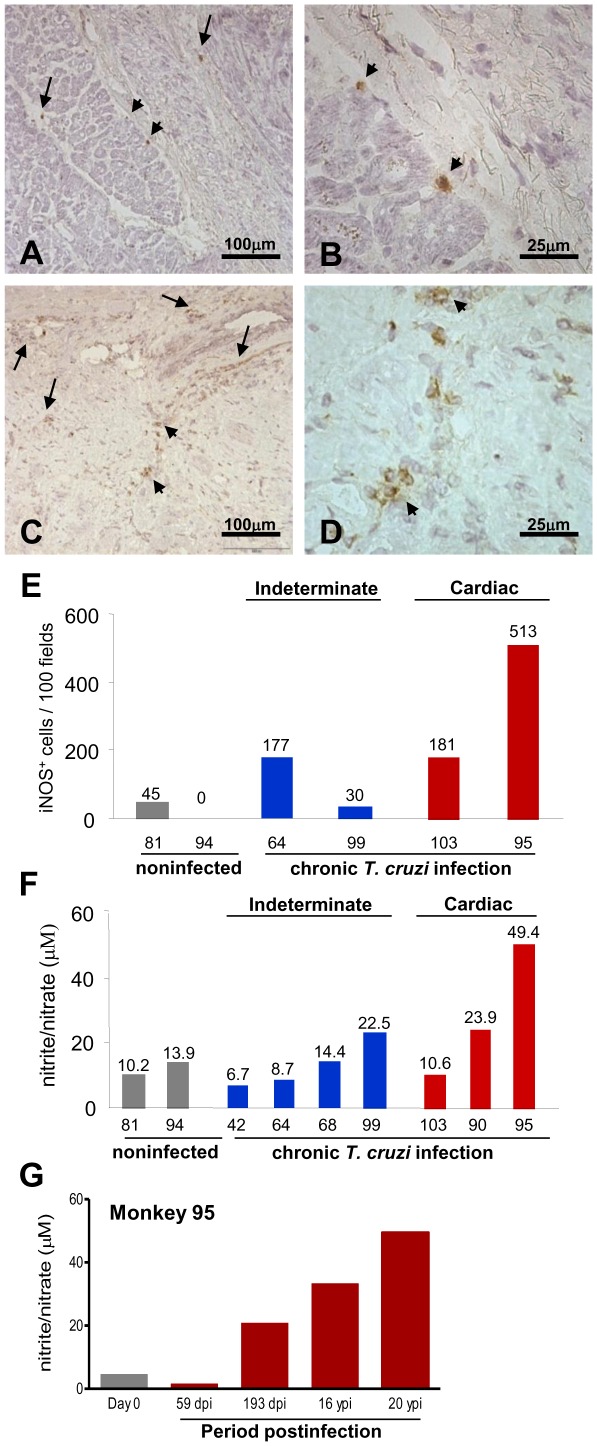
iNOS/NOS2^+^ cells in the myocardium and NO in the serum of *T. cruzi*-infected rhesus monkeys. The presence of iNOS/NOS2^+^ cells in the myocardium of the left ventricles was immunohistochemically detected and NO concentration was evaluated by a Griess-based method. Photomicrograph of myocardium section of the left ventricle (**A**) and (**B**) of the noninfected monkey #81, which showed a few iNOS/NOS2^+^ cells. Photomicrograph of myocardioum section of the left ventricle (**C**) and (**D**) of the *T. cruzi*-infected monkey #95 (20 ypi). (**E**) Number of iNOS/NOS2^+^ cells in 100 microscopic fields in heart sections. (**F**) Concentration of NO in the serum of noninfected controls and chronically (20–23 ypi) *T. cruzi*-infected monkeys. (**G**) Concentration of NO in the serum of monkey #95 prior to infection (day 0), during the acute phase when parasitemia was positive (56 dpi) and negative (163 dpi) and during the chronic phase (16 ypi and 20 ypi). Bar = 100 µm in (**A**) and (**C**); Bar = 25 µm in (**B**) and (**D**).

Because parasite antigens were scarce in the cardiac tissues of monkeys with the indeterminate and cardiac forms of Chagas disease, we evaluated the cardiac tissue for the expression of iNOS/NOS2, which is an enzyme potentially involved in an important parasite control pathway [9], [11]. Analysis of serial heart tissue sections revealed that most of the iNOS/NOS2^+^ cells infiltrating the myocardial interstitium of infected animals (Figure 3A–D) were CD68^+^ macrophages (data not shown). There was a significant increase in the number of iNOS/NOS2^+^ cells in the myocardium of monkey #95 that presented high parasitism detected by qPCR for genomic DNA (Figure 3A–D, Figure 3E), whereas iNOS/NOS2^+^ cells were scarce or absent in noninfected animals (Figure 3E).

In chronic Chagas disease, severity of CCC correlated with high NO levels in serum [6]. Because an increased number of iNOS/NOS2^+^ cells in the cardiac tissue of infected monkeys was related to the severity of electrical abnormalities, we decided to study NO levels in the serum of infected monkeys. A high NO concentration was only detected in the monkey (#95) with severe CCC (Figure 3F). In this monkey, parasite control was achieved at 2 months postinfection, at which point antibodies were detected (Figure 2G), in absence of significant NO concentration in serum (Figure 3G). A high NO concentration was only detected after 6 months of infection, and the NO level was persistently elevated at 16 and 20 ypi (Figure 3G). In the case of noninfected monkeys #81 and #94 the low numbers of iNOS/NO2^+^ cells in the cardiac tissue was parallel to low NO levels in serum (Figure 3E, Figure 3F). In the cardiopatic *T. cruzi*-infected monkey #95 the high number of iNOS/NO2^+^ cells in the cardiac tissue paralleled the high NO concentration in serum (Figure 3E, Figure 3F). However, there was no significant correlation (r^2^ = 0.5206, p = 0.105) between the number of iNOS/NO2^+^ cells in the cardiac tissue and NO concentration in serum of *T. cruzi*-infected rhesus monkeys.

### Cardiac tissue injury is severe in *Trypanosoma cruzi*-infected monkeys overexpressing iNOS/NOS2 and NO

**Figure 4:**
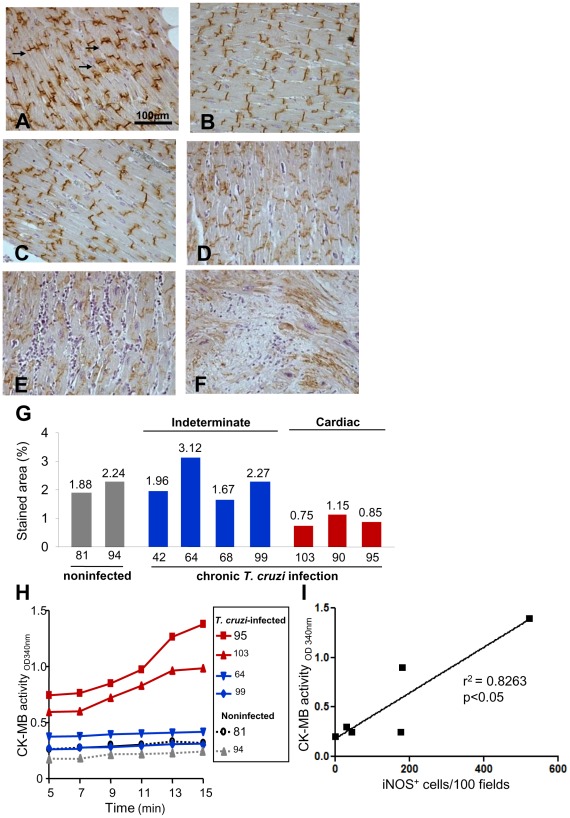
Cardiomyocyte injury in *T. cruzi*-infected rhesus monkeys. Cardiomyocyte damage was assessed by immunohistochemical detection of Cx43 in the myocardium of the left ventricle and CK-MB activity levels in the serum of noninfected and chronically *T. cruzi*-infected rhesus monkeys. (**A**) Photomicrograph of myocardium section of the left ventricle of the noninfected monkey #94 showing normal pattern of Cx43 expression in intercalated discs. (**B**) Photomicrograph of myocardium section of the left ventricle of the *T. cruzi*-infected monkey #64 (23 ypi) showing normal aspect. (**C**) Photomicrograph of left ventricle section of the *T. cruzi*-infected monkey #99 (20 ypi) showing normal Cx43 pattern. (**D**) Photomicrograph of section of left ventricle of the *T. cruzi*-infected monkey #90 (20 ypi) revealing Cx43 loss in myocardial area lacking inflammation. (**E–F**). Photomicrographs of left ventricle section of the cardiopatic *T. cruzi*-infected monkey #95 (20 ypi) showing Cx43 loss in area with (**E**) intense diffuse inflammation and (**F**) the substitution of cardiomyocytes by mesenchymal cells. (**G**) Frequency of stained Cx43 area in heart sections of noninfected and chronically *T. cruzi*-infected monkeys (20–23 ypi). (**H**) Detection of CK-MB activity in the serum of noninfected and chronically *T. cruzi*-infected monkeys (20–23 ypi). (**I**) Correlation between the number of iNOS/NOS2^+^ cells in heart tissue and CK-MB activity levels in serum of rhesus monkeys. Bar = 100 µm.

Overexpression of iNOS/NOS2 and NO has been shown to be associated with heart injury in noninfectious conditions [8]. To test the possible consequences of iNOS/NOS2 overexpression in the heart tissue of *T. cruzi*-infected monkeys, we analyzed the expression of Cx43. Connexin 43 is the major gap junction protein in the heart, and Cx43 is primarily responsible for the electrical synchrony of cardiomyocytes [21]. Connexin 43 distribution was homogeneous in the intercalated disks of cardiomyocytes of noninfected controls (Figure 4A). Further, the Cx43 pattern and stained area in the indeterminate *T. cruzi*-infected monkeys 64 (Figure 4B) and #99 (Figure 4C) were preserved, and the Cx43 staining resembled the noninfected controls (Figure 4G). Conversely, Cx43 was detected as disorganized patches scattered throughout the tissue of cardiopatic infected monkeys #90 (Figure 4D), #95 (Figure 4E, Figure 4F) and #103 (data not shown). In addition, Cx43 loss was associated with inflammatory infiltrates (Figure 4E, Figure 4F). Although not considered for the quantitative study, in cardiac areas where myocardial cells were substituted by mesenchymal cells Cx43 loss was, obviously, more pronounced (Figure 4F). Monkey #95, which showed the more severe form of CCC and a high number of iNOS/NOS2^+^ cells in the heart, also exhibited a marked Cx43 loss (Figure 4E, Figure 4F, Figure 4G). Interestingly, when the percentage of Cx43-stained area in the cardiac tissue was evaluated considering the clinical score, significant Cx43 loss was detected in cardiopatic *T. cruzi*-infected monkeys (0.92±0.12% of stained area) when compared with indeterminate (2.25±0.31% of stained area; p<0.05) and noninfected (2.06±0.18% of stained area; p<0.05) rhesus monkeys. Increased CK-MB activity levels in serum, which is a marker of myocardial cell injury [22], was detected in cardiopatic monkeys #95 and #103 (Figure 4H). In the infected monkeys with normal ECG patterns (#64 and #99), the CK-MB activity levels resembled the levels in the noninfected controls (#81 and #94). A positive correlation was seen between the Cx43 loss in the cardiac tissue and the CK-MB activity levels in serum of the studied rhesus monkeys (r^2^ = 0.6355; *p*<0.05). Importantly, there was a positive correlation between the number of iNOS/NOS2^+^ cells in the heart tissue and the CK-MB activity level in the serum (r^2^ = 0.8263; *p*<0.05) of chronically *T. cruzi*-infected rhesus monkeys (Figure 4I).

### Decreased myocardial cell lesion and heart injury in iNOS/NOS-deficient mice

**Figure 5:**
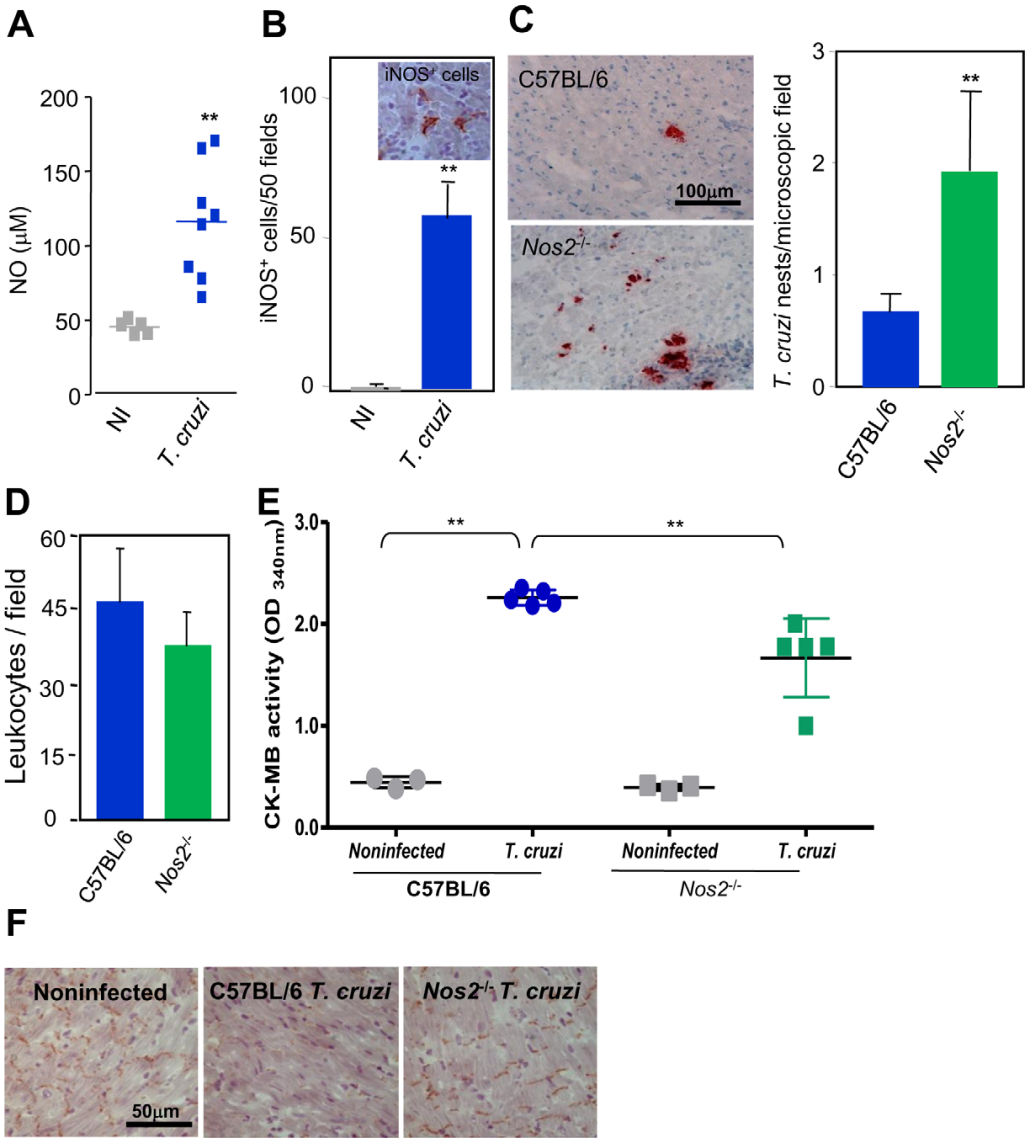
iNOS/NOS2 and NO status influence heart parasitism and cardiomyocyte integrity in *T. cruzi*-infected mice. The mice were infected with 100 blood trypomastigotes of the Colombian *T. cruzi* strain and analyzed at 40 dpi. The presence of iNOS/NOS2^+^ cells, parasite nests, inflammatory cells and Cx43 in the myocardium was immunohistochemically detected, NO concentration was evaluated by a Griess-based method and CK-MB activity levels in the serum was biochemically determined. (**A**) Increased NO levels in serum of *T. cruzi*-infected C57BL/6 mice in comparison with noninfected controls (NI). (**B**) Photomicrograph of iNOS/NOS2^+^ cells in the cardiac tissue of infected C57BL/6 mice and quantification of iNOS/NOS2^+^ cells in the cardiac tissue of infected C57BL/6 mice in comparison with noninfected controls. (**C**) Photomicrographs and quantification of parasite nests showing increased heart parasitism in *Nos2* ^−/−^ compared with *T. cruzi*-infected C57BL/6 mice. (**D**) Similar number of inflammatory cells in the heart tissue of C57BL/6 and *Nos2* ^−/−^ *T. cruzi*-infected mice. (**E**) CK-MB activity levels in the serum of noninfected and *T. cruzi*-infected mice revealing increased CK-MB activity in *T. cruzi*-infected mice when compared with noninfected controls. Decreased CK-MB activity in the serum of *Nos2* ^−/−^ compared with C57BL/6 *T. cruzi*-infected mice. (**F**) Preserved expression of Cx43 in the heart tissue of *Nos2* ^−/−^ compared with C57BL/6 infected mice. Analysis at 40 dpi of 3–5 noninfected and 5–8 infected mice/group. * *p*<0.05 and ** *p*<0.01. Bar = 100 µm (**C**). Bar = 50 µm (**F**).

To add insights on the participation of iNOS/NOS2-derived NO in *T. cruzi*-elicited cardiomyopathy, we studied the effect of the infection of iNOS/NOS2-deficient (*Nos2* ^tm1Lau^/J) mice in different aspects of heart injury. After 40 days of infection, in comparison with noninfected controls wild-type C57BL/6 mice present increased NO levels in serum (Figure 5A), which are not detected in *Nos2* ^−/−^ mice (4.4±0.17 µM in noninfected *vs.* 4.3±2.1 µM in *T. cruzi*-infected). In addition, there was an increased number of iNOS/NOS2^+^ cells in the cardiac tissue of *T. cruzi*-infected C57BL/6 mice (Figure 5B). Interestingly, a significant increase (*p*<0.05) in the number of parasite nests was seen in the cardiac tissue of *Nos2* ^−/−^ mice (Figure 5C). Further, a similar intensity of myocardial inflammation (Figure 5D) and frequency of ICAM-1^+^ and VCAM-1^+^ blood vessels (data not shown) were detected in wild-type and *Nos2* ^−/−^ mice. Representative ECG registers at 40 dpi are shown in the Figure S4. Absence of iNOS/NOS2 led to sinus bradyarrhythmia in noninfected controls compared with noninfected wild-type C57BL/6 mice. Further, *T. cruzi*-infected C57BL/6 and *Nos2* ^−/−^presented increased PR interval, resulting in first-degree AV block (Table 2). Importantly, *T. cruzi*-infected mice showed concomitantly two types of arrhythmia, sinus bradycardia and AV block. Significant increase in QTc and higher QRS scores were detected in *T. cruzi*-infected C57BL/6 mice compared with noninfected mice (Table 2). Interestingly, changes in QRS scores were significantly decreased in infected *Nos2* ^−/−^ mice compared with infected C57BL/6 mice (Table 2, *p*<0.001). Altogether, *T. cruzi* infection resulted in more frequent first- and second- degree atrioventricular block type 2∶1 in infected C57BL/6 mice compared with infected *Nos2* ^−/−^ mice (Table 2). Considering the importance of myocardial cell integrity for electrical conduction and the participation of Cx43 in this process (21), we analyzed the CK-MB levels in serum and Cx43expression in the cardiac fibers of iNOS/NOS2 deficient *T. cruzi*-infected mice. Importantly, when compared with infected C57BL/6 mice, *Nos2* ^−/−^ mice had a significant decrease in CK-MB activity levels in serum (Figure 5E), although myocardial cell lesion was not completely abrogated. Additionally, absence of iNOS/NOS2 resulted in inhibition of Cx43 loss in the cardiac tissue of *T. cruzi*-infected mice (Figure 5F). Taken together, these data reveal decreased cardiomyocyte lesion and more preserved heart conduction and ventricle polarization in *T. cruzi*-infected mice with iNOS/NOS2 deficiency.

**Table 2:** Electrocardiograph parameters of C57BL/6 and *Nos2* ^−/−^ mice infected with the Colombian *T. cruzi* strain.

Experimental groups	Heart rate† (bpm)	PR interval (ms)	P-wave duration (ms)	QRS duration (ms)	QTc (ms)	Cardiac conduction (% of mice)‡
C56BL/6 NI	530±28.1	35.8±2.1	12.3±1.4	11.1±0.4	16.3±2.2	Brad (0%) AVB1 (0%) AVB2 (0%)
C57BL/6 *T. cruzi*	441±38.5**	41.1±0.4***	12.5±0.9	18.6±1.5***	32.2±2.7***	Brad (43%) AVB1 (57%) AVB2 (43%)
*Nos2* ^−/−^ NI	400±63.8§§	36.5±4.0	14.0±1.0	11.1±1.1	20.7±1.7§§	Brad (60%) AVB1 (20%) AVB2 (0%)
*Nos2* ^−/−^ *T. cruzi*	423±81.9	39.6±4.3	14.0±2.3	13.9±2.4* ^,^ ###	28.3±9.8	Brad (57%) AVB1 (43%) AVB2 (28%)

**†:** ECG parameters were evaluated at 40 dpi, using the following standard criteria: (i) heart rate (monitored by beats per minute (bpm), and (ii) the variation of the P wave and PR, QRS and QT intervals, all measured in milliseconds (ms); Brad, bradycardia; AVB1, first-degree atrioventricular block; AVB2, second-degree atrioventricular block.

**‡:** These data represent two independent experiments, with 5–7 mice/group.

**§:** , p<0.05;

**§§:** , p<0.01; comparison between the values for C57BL/6 and *Nos2* ^−/−^ noninfected groups of mice;

*: , p<0.05;

**: , p<0.01;

***: , p<0.001; comparison between the values for noninfected and *T. cruzi*-infected groups of mice;

# , p<0.05;

## , p<0.01;

### , p<0.001; comparison between the values for C57BL/6 and *Nos2* ^−/−^ *T. cruzi*-infected mice.

## Discussion

In the present study, the infection of nonhuman primate rhesus monkeys with the Colombian *T. cruzi* strain reproduced clinical and histopathological aspects of the chronic indeterminate and cardiac forms of Chagas disease, including parasite persistence in the cardiac tissue. Moreover, overexpression of iNOS/NOS2 cells in the cardiac tissue and systemic high NO levels were directly related to cardiomyocyte lesion and heart injury, including electrical abnormalities. Studies in *Nos2* ^−/−^ mice corroborated the participation of iNOS/NOS2 in parasite dissemination control and, moreover, revealed the participation of the iNOS/NOS2-derived NO in disturbance of the major molecular pathway involved in electrical synchrony in *T. cruzi* infection.

The reproduction of several features of CCC, which is the main clinical manifestation of Chagas disease, in *T. cruzi*-infected rhesus monkeys allows the adoption of this model to test vaccines and new trypanocide chemotherapies. In addition, *T. cruzi*-infected rhesus monkeys can be used to investigate mechanisms of CCC physiopathology [1]. Although the outbred nature and small number of monkeys can be viewed as a limitation of the present studies, these animals reproduced the clinical spectrum (indeterminate and cardiac form) seen in Chagas disease [2], [19]. Moreover, the chronically infected monkeys reproduced the most typical chagasic electrical conduction abnormalities [2]. Although no increase in the cardiothoracic index was noticed in infected monkeys, asynchronic interventricular septum motility was detected in monkey #95, which showed a more severe stage of CCC. Electrical conduction alterations were associated with the intensity of myocardial fibrosis, which was spatially related to inflammation in an apparent lack of parasite and resembled the features of Chagas disease [2], [19]. Myocardial fibrosis was directly related to the 12-lead ECG QRS scoring and the severity of Chagas' heart disease [23]. Fibrosis, which is one of the most important features of CCC [19], shows a progressive evolution in *T. cruzi*-infected mice [24]. Fibrosis is directed by inflammatory processes that provoke chemokine-driven accumulation of mesenchymal cells [25]. In the present studies, collagen deposition occurred early in the acute infection (41–76 dpi) concomitantly with inflammation. Interestingly, inflammation resolution and collagen degradation can occur without specific treatment. This finding was seen in monkey #45, which presented the indeterminate form of Chagas disease at 3 ypi. Monkey #99 did not exhibit cardiac inflammation or fibrosis; however, we could not prove whether fibrosis was established and later remodeled. The predominance of type III, pro-III, and pro-IV collagens in the heart tissue of individuals with chronic *T. cruzi* infection [24] may favor reversibility of fibrosis because these collagens have a high turnover. In *T. cruzi* infection, extracellular matrix deposition in the cardiac tissue can be remodeled if inflammation subsides, which has been observed after etiological chemotherapy with benznidazole in the chronic infection [24] and after modulation of inflammation with a partial CC-chemokine receptor antagonist in the acute and chronic infection [26], [27]. These data suggest parasites and inflammation as triggers of fibrogenic factors in CCC, which reinforces the idea that therapeutic strategies targeting parasites and inflammation, possibly combined, may be beneficial in remodeling fibrosis and restoring heart function in Chagas disease.

Observations of chronic myocarditis and fibrosis in the apparent absence of *T. cruzi* have suggested that autoimmunity is a central mechanism for CCC pathogenesis [3]. In a classic study, however, amastigote forms of *T. cruzi* were detected inside myocardial cells in all analyzed CCC patients [19]. Furthermore, a PCR signal for *T. cruzi* kDNA has been observed in the hearts of CCC patients, but it was not detected in seropositive nonCCC patients [28]. In indeterminate and cardiopatic *T. cruzi*-infected monkeys, parasites (antigen, kDNA and nuclear satellite DNA) persisted in the heart and spleen. Although in some tissue samples parasite kDNA was not detected in repeated analyses, the study of different fragments of heart and spleen and the use of more sensitive assay resulted in detection of parasite persistence in all chronically *T. cruzi*-infected rhesus monkeys. Considering the short life span of parasite DNA in host tissues [29], PCR signals in the heart and spleen of infected monkeys constitute real proof of *T. cruzi* persistence. The presence of anti-*T. cruzi* antibodies in cardiopatic monkeys coincided with parasite persistence in the blood, heart and spleen. In monkey #99, however, anti-*T. cruzi* antibodies were restricted to the acute infection and coincided with parasite control. Detection of a specific immune response in chronically infected individuals reflects continuous antigenic stimulus by persistent parasites. The consistent absence of detection of specific antibodies [15], *T. cruzi* kDNA in peripheral blood and heart in monkey #99, which had no electrical abnormalities, cardiac inflammation or fibrosis at 20 ypi in the absence of treatment, led us to consider the possibility of spontaneous cure in this monkey [19]. However, the analysis of different fragments of spleen and heart revealed the presence of *T. cruzi* genomic DNA in both tissues of monkey #99, supporting the persistence of parasite in these tissues in this monkey with the indeterminate form of Chagas disease. Therefore, the low parasitism restricted to focal areas in different organs may explain the difficulties to reveal parasite and antibodies presence in this monkey.

Although there was no correlation between the quantity of antigens and the intensity of myocarditis, the detection of *T. cruzi* DNA in the cardiac tissue of infected monkeys supports the idea that persisting parasites trigger detrimental inflammation that can act on cardiomyocytes. Heart inflammation is a major factor that contributes to an increased risk of death in CCC compared with other heart conditions [30]. The components of inflammatory infiltrates contributing to this picture remain unclear. In noninfectious human diseases and murine models of cardiac pathologies, iNOS/NOS2 and NO were shown to be both protective and detrimental for heart physiology, and these effects may be dependent on NO concentration [8], [31]. Therefore, the increased number of iNOS/NOS2^+^ cells in the heart tissue of *T. cruzi*-infected cardiopatic monkeys led us to search for cardiomyocyte lesion. Connexin 43 was severely depleted in cardiopatic monkey #95, which presented a high number of iNOS/NOS2^+^ cells infiltrating the cardiac tissue. *T. cruzi* infection of human cardiomyocytes *in vitro* [32] and mice *in vivo* [27], [33] has also been shown to cause Cx43 loss. This effect was hampered in infected TNFR1-deficient *T. cruzi*-infected mice, which supports that TNF/TNFR1 signaling is involved in Cx43 loss [33]. Tumor necrosis factor, particularly associated with IFNγ, is an iNOS/NOS2 inducer that results in NO production [7], [8]. In this context, we detected IFNγ in the serum of monkey #95 (150 pg/mL) and TNF in the serum of monkey #103 (28 pg/mL) and high IFNγ and TNF levels in PMA-stimulated peripheral blood cells of monkeys #103 and #95, but not in noninfected controls or infected monkeys with the indeterminate form of Chagas disease (our unpublished data). Although consistent with the idea that the cardiopatic monkeys #103 and #95 overproduce inflammatory cytokines that may stimulate NO production, the low number of responsive animals hampered any definitive conclusion. Thus, the mechanism by which high iNOS/NOS2 is induced in cardiac tissue following *T. cruzi* infection remains unknown. The present study was the first to show that in CCC the numbers of iNOS/NOS2^+^ cells in cardiac tissue are associated with Cx43 loss and, more clearly, with increased CK-MB activity levels in serum, markers of cardiomyocyte injury [21], [22]. Interestingly, iNOS/NOS2 upregulation in an inflammatory milieu results in increased NO levels in the cardiac tissue in autoimmune myocarditis [34]. In addition, NO is induced in macrophages and cardiomyocytes by *T. cruzi* and inflammatory cytokines and chemokines, which require the iNOS/L-arginine pathway [35]. The severity of CCC is associated with high NO levels in chagasic patients [6]. Nitric oxide has also been shown to be involved in heart denervation in acute *T. cruzi* infection in rats [31]. Therefore, persistent *T. cruzi* in the cardiac tissue might sustain continuous iNOS/NOS2 expression and a large supply of NO in a tissue that normally experiences low and tightly controlled levels of this mediator [7], [36]. In consequence, the increased expression of iNOS/NOS2 and supply of NO could lead to cardiomyocyte lesion and heart injury. To test this idea, we adopted a *Nos2* ^−/−^ murine model. Initially, *T. cruzi* infection of C57BL/6 mice (the *Nos2* ^−/−^ genetic background) led to increased systemic NO production and, particularly, iNOS/NOS2^+^ cells in the cardiac tissue, which reproduced our findings in chronically infected monkeys. In addition, iNOS/NOS2^+^ cells have previously been detected in the cardiac tissue of acutely infected mice [9] and dogs [37]. Therefore, the present data show that independent of the host and the phase of infection, *T. cruzi* infection enhances NO in serum and iNOS/NOS2^+^ cells in the cardiac tissue. The present data also refute the result showing that iNOS/NOS2 is not required for *T. cruzi* control [12], but support previous findings that iNOS/NOS2 is essential for *T. cruzi* control in the cardiac tissue [9], [11]. Paradoxically, NO might be detrimental in *T. cruzi* infection because it depresses lymphocyte functions, which could promote parasite survival [7]. Although this idea demands further experimental support, if this is the case the detrimental effect of NO on the immune response may explain parasite persistence in chronically infected individuals, including patients [6] and our experimental models. Interestingly, iNOS/NOS2 absence (in *Nos2* ^−/−^) abolished NO production in *T. cruzi*-infected mice, which showed that iNOS/NOS2 is essential for NO overproduction. In addition, this result corroborated studies showing that there is no compensation mechanism increasing other NOS isoforms in Chagas disease [12]. Furthermore, iNOS/NOS2 and locally produced NO are not involved in myocarditis formation, which suggests that the nature rather than the intensity of heart inflammation is determinant of the Chagas' heart disease outcome [27]. Furthermore, *T. cruzi* infection of *Nos2* ^−/−^ mice demonstrated a role for iNOS/NOS2 in myocardial cell lesion and connectivity loss, which supported our findings in chronically infected monkeys. Interestingly, recent proposal brings support for Chagas disease to be considered a junctionopathy [38]. Therefore, iNOS/NOS2-derived NO may be a direct or indirect critical trigger of the molecular pathway leading to myocardial cell connectivity loss. Further, *T. cruzi* may directly lead to myocardial cell lesion, as revealed by increased CK-MB activity levels in serum and Cx43 loss, in infected mice. In this context, absence of iNOS/NOS2 led to significant decrease in CK-MB activity levels in serum, placing iNOS/NOS2-derived NO as important myocardiotoxic agent, but did not completely abolished myocardial cell lesion that persisted in presence of high cardiac tissue parasitism in acutely *T. cruzi*-infected *Nos2* ^−/−^ mice. In chronically infected rhesus monkeys, focal persistence of *T. cruzi* (revealed by detection of antigen^+^ spots and of low amounts of parasite DNA) may contribute to maintain iNOS/NOS2 induction and local NO production leading to myocardial cell lesion. A previous study showed that iNOS/NOS2-derived NO was associated with right ventricular dilation and systolic dysfunction in acute murine *T. cruzi*-elicited myocarditis [9]. Although NOS/NOS2 deficiency led to bradyarrhythmia in noninfected controls, *T. cruzi*-infected *Nos2* ^−/−^ mice presented lower frequency of AVB1 and AVB2 than *T. cruzi*-infected C57BL/6 mice. More importantly, the higher QRS scores detected in CCC patients [2], 23, which were reproduced in infected monkeys and C57BL/6 mice, were significantly decreased in *T. cruzi*-infected *Nos2* ^−/−^ mice, which implicated iNOS/NOS2 and NO in QRS score increases independent of the host. Therefore, the present data support that the iNOS/NOS2-NO pathway participates in *T. cruzi*-induced myocardial cell lesion and heart injury and suggest that this pathway should be explored as a therapeutic target in CCC. Considering observations in chronic Chagas disease [6], it is a plausible proposal as the pivotal role of NO in *T. cruzi* control was restricted to acute infection [39]. Further studies are needed to determine whether inhibition of iNOS/NOS2 will be therapeutically useful in chronic Chagas disease, a condition of vast overproduction of NO.

## Supporting Information

Figure S1 **Representative electrocardiographic registers of chronically** ***T. cruzi*** **-infected rhesus monkeys.** Rhesus monkeys were infected with metacyclic trypomastigotes of the Colombian *T. cruzi* strain and analyzed at 20–23 years post-infection (ypi). (**A**) ECG registers of monkey # 64 (23 ypi) showing normal pattern of electrical activity. (**B**) ECG registers of monkey # 99 (20 ypi) showing normal pattern. (**C**) ECG registers of monkey # 103 (20 ypi) demonstrating first degree right bundle branch block (RBBB1, arrow). (**D**) ECG registers of monkey # 90 (20 ypi) showing first degree left bundle branch block (LBBB1, arrow). (**E**) ECG registers of monkey # 95 (20 ypi) showing second degree right bundle branch block (RBBB2, arrow) and right QRS axis deviation (RAD, arrow head).
(TIF)

Figure S2 **Histological alterations in the myocardium of** ***T. cruzi*** **-infected rhesus monkeys.** Sections of the myocardium of the left ventricle of noninfected and *T. cruzi*-infected rhesus monkeys were stained with H&E and analyzed under light microscope. Photomicrographs of heart tissue sections showing: (**A**) Monkey #42 (20 ypi), with multifocal infiltrates of mononuclear inflammatory cells. (**B**) Monkey #64 (23 ypi), intense focal mononuclear inflammation. (**C**) Monkey #99 (20 ypi), normal aspect of the myocardium. (**D**) Monkey #103 (20 ypi), multifocal infiltrates of mononuclear inflammatory cells. (**E**) Monkey #90 (20 ypi), normal aspect of the myocardium. (**F**) Monkey #95 (20 ypi), multifocal infiltrates of mononuclear inflammatory cells. (**G**) Noninfected monkey #94, normal aspect. H&E. Bar = 100 µm.
(TIF)

Figure S3 **Total IgM and IgG levels in noninfected and chronically** ***T. cruzi*** **-infected rhesus monkeys.** The levels of total immunoglobulins of the IgM and IgG classes were determined by nephrolometry in the serum of noninfected and *T. cruzi*-infected rhesus monkeys at the end point (20–23 years post-infection, ypi). Total (**A**) IgM and (**B**) IgG levels in the serum.
(TIF)

Figure S4 **Representative electrocardiographic registers of** ***T. cruzi*** **-infected C57BL/6 and** ***Nos2*** **^−/−^ mice.** The mice were infected with 100 blood trypomastigotes of the Colombian *T. cruzi* strain and analyzed at 40 dpi. (**A**) ECG registers of noninfected control C57BL/6 mice showing normal pattern of electrical activity. (**B**) ECG registers of *T. cruzi*-infected C57BL/6 mice showing first- and second-degree atrioventricular block (AVB1, AVB2, arrows). (**C**) ECG registers of noninfected control *Nos2* ^−/−^ mice showing presence of sinus bradyarrhythmia. (**D**) ECG registers of *T. cruzi*-infected *Nos2* ^−/−^ mice showing first degree atrioventricular block (AVB1, arrows).
(TIF)

Table S1 **General characterization of** ***Trypanosoma cruzi*** **infection of rhesus monkeys.** Rhesus monkeys were infected with metacyclic trypomastigote forms of the Colombian *T. cruzi* strain and analyzed at 20–23 years post-infection (ypi). The original colony identification used in the present study and the experimental number used in a previous publication that describes the analysis of the *T. cruzi*- infected monkeys during the acute infection, are provided.
(DOC)

Table S2 **Echocardiographic patterns detected in** ***Trypanosoma cruzi*** **-infected rhesus monkeys during chronic infection.** Rhesus monkeys were infected with metacyclic trypomastigotes of the Colombian *T. cruzi* strain and analyzed at 20–23 years post-infection (ypi). The echocardiographic registers were analyzed and the main findings were asynchronic interventricular septum motility and decreased left ventricular ejection fraction detected in monkey #95.
(DOC)
