# Supplementary figures and images for: Inducible Nitric Oxide Synthase in Heart Tissue and Nitric Oxide in Serum of Trypanosoma cruzi-Infected Rhesus Monkeys: Association with Heart Injury
Source: PLoS Negl Trop Dis. 2012 May 8;6(5):e1644. doi: 10.1371/journal.pntd.0001644 (PMC3348164; doi:10.1371/journal.pntd.0001644)

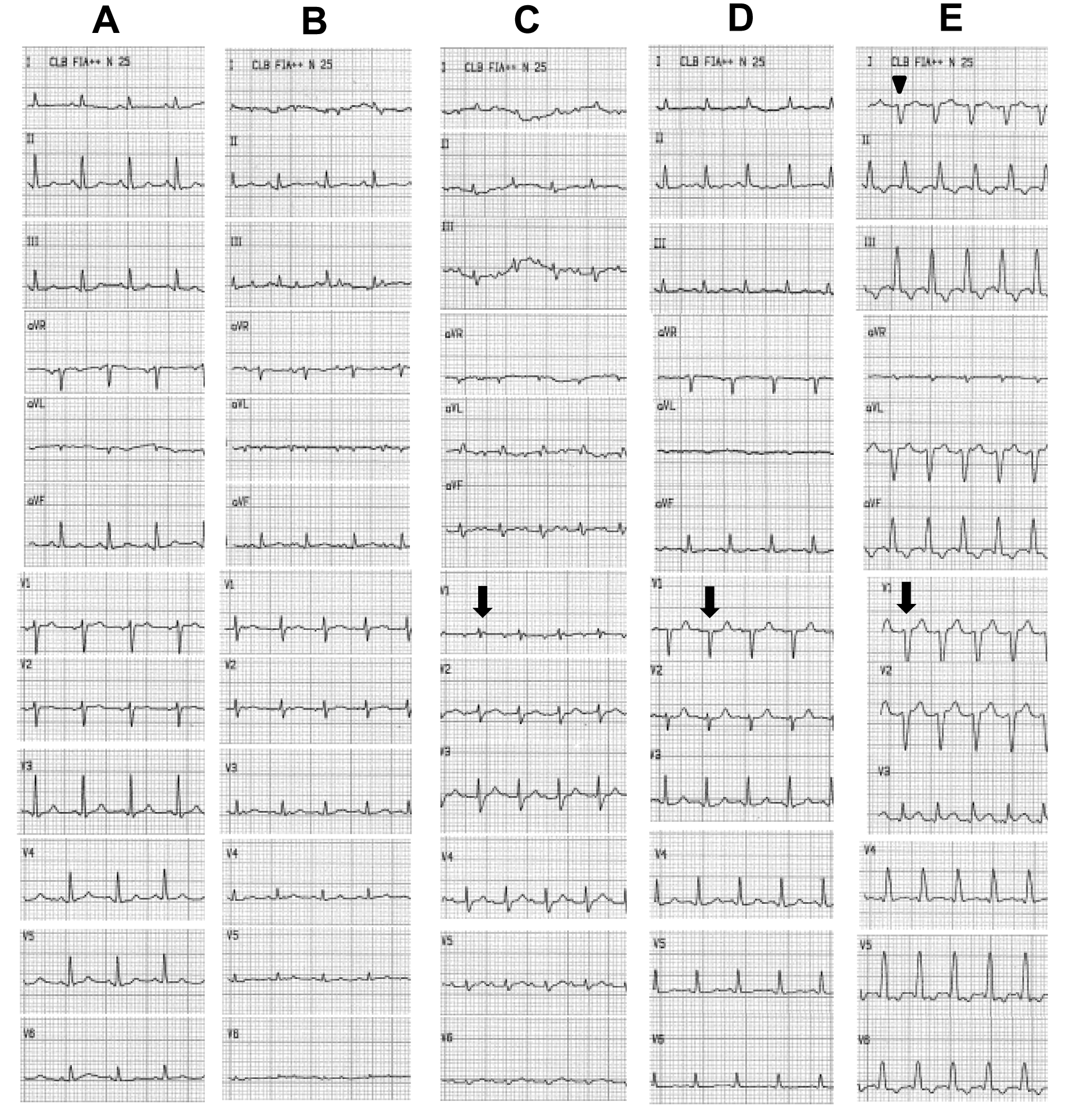

Supplement: Figure S1 — Representative electrocardiographic registers of chronically T. cruzi -infected rhesus monkeys. Rhesus monkeys were infected with metacyclic trypomastigotes of the Colombian T. cruzi strain and analyzed at 20–23 years post-infection (ypi). (A) ECG registers of monkey # 64 (23 ypi) showing normal pattern of electrical activity. (B) ECG registers of monkey # 99 (20 ypi) showing normal pattern. (C) ECG registers of monkey # 103 (20 ypi) demonstrating first degree right bundle branch block (RBBB1, arrow). (D) ECG registers of monkey # 90 (20 ypi) showing first degree left bundle branch block (LBBB1, arrow). (E) ECG registers of monkey # 95 (20 ypi) showing second degree right bundle branch block (RBBB2, arrow) and right QRS axis deviation (RAD, arrow head). (TIF) [file pntd.0001644.s001.tif]

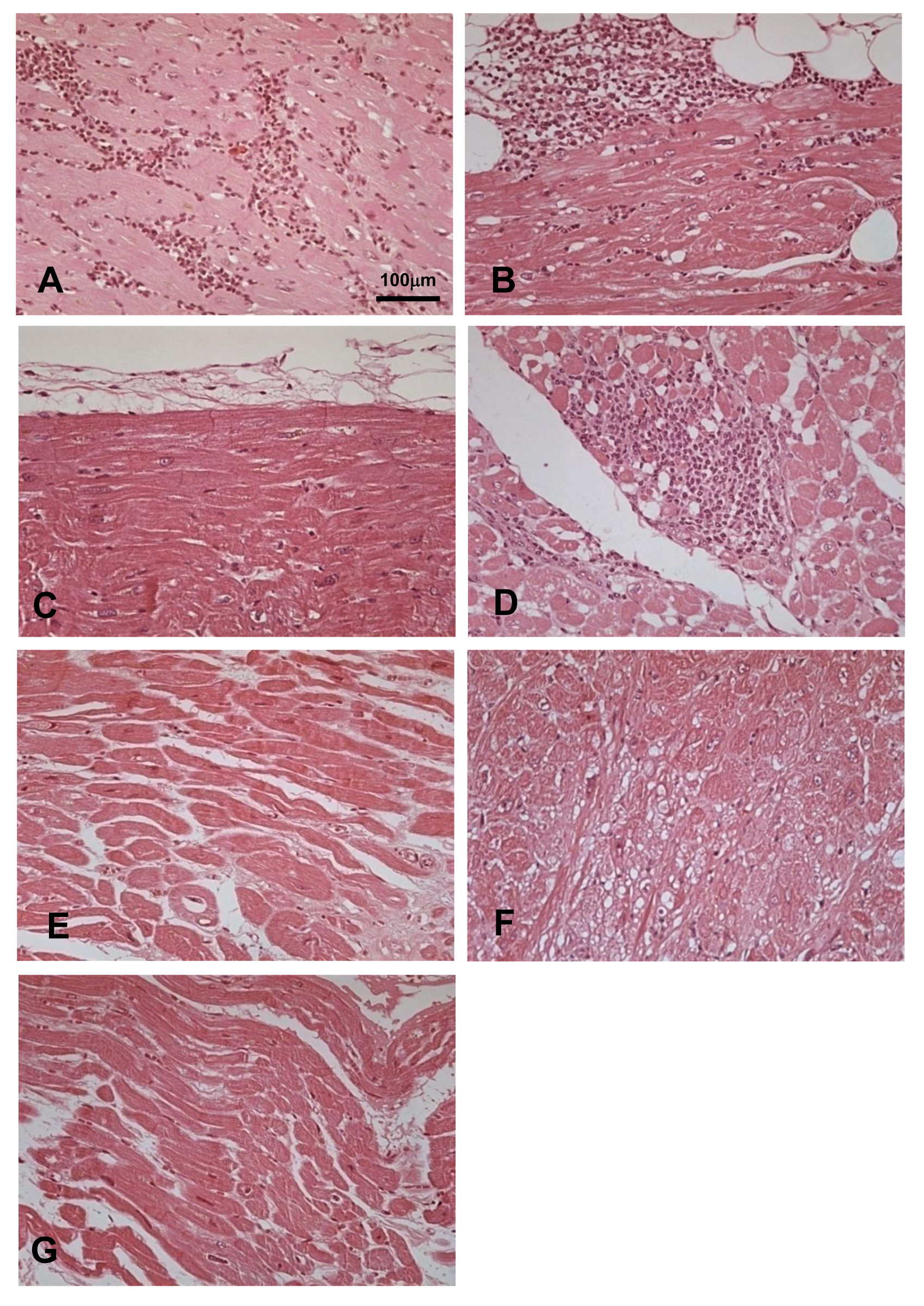

Supplement: Figure S2 — Histological alterations in the myocardium of T. cruzi -infected rhesus monkeys. Sections of the myocardium of the left ventricle of noninfected and T. cruzi-infected rhesus monkeys were stained with H&E and analyzed under light microscope. Photomicrographs of heart tissue sections showing: (A) Monkey #42 (20 ypi), with multifocal infiltrates of mononuclear inflammatory cells. (B) Monkey #64 (23 ypi), intense focal mononuclear inflammation. (C) Monkey #99 (20 ypi), normal aspect of the myocardium. (D) Monkey #103 (20 ypi), multifocal infiltrates of mononuclear inflammatory cells. (E) Monkey #90 (20 ypi), normal aspect of the myocardium. (F) Monkey #95 (20 ypi), multifocal infiltrates of mononuclear inflammatory cells. (G) Noninfected monkey #94, normal aspect. H&E. Bar = 100 µm. (TIF) [file pntd.0001644.s002.tif]

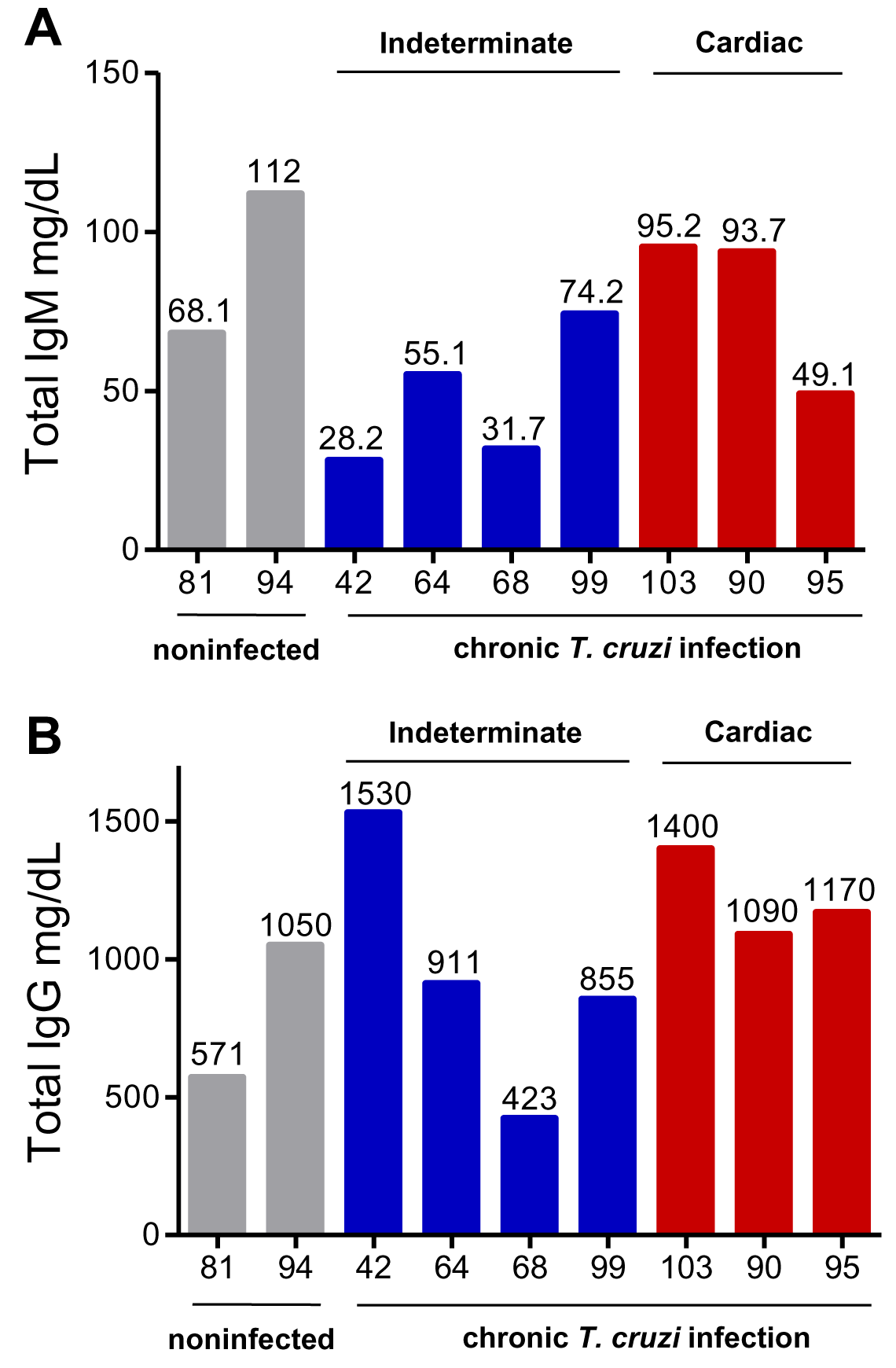

Supplement: Figure S3 — Total IgM and IgG levels in noninfected and chronically T. cruzi -infected rhesus monkeys. The levels of total immunoglobulins of the IgM and IgG classes were determined by nephrolometry in the serum of noninfected and T. cruzi-infected rhesus monkeys at the end point (20–23 years post-infection, ypi). Total (A) IgM and (B) IgG levels in the serum. (TIF) [file pntd.0001644.s003.tif]

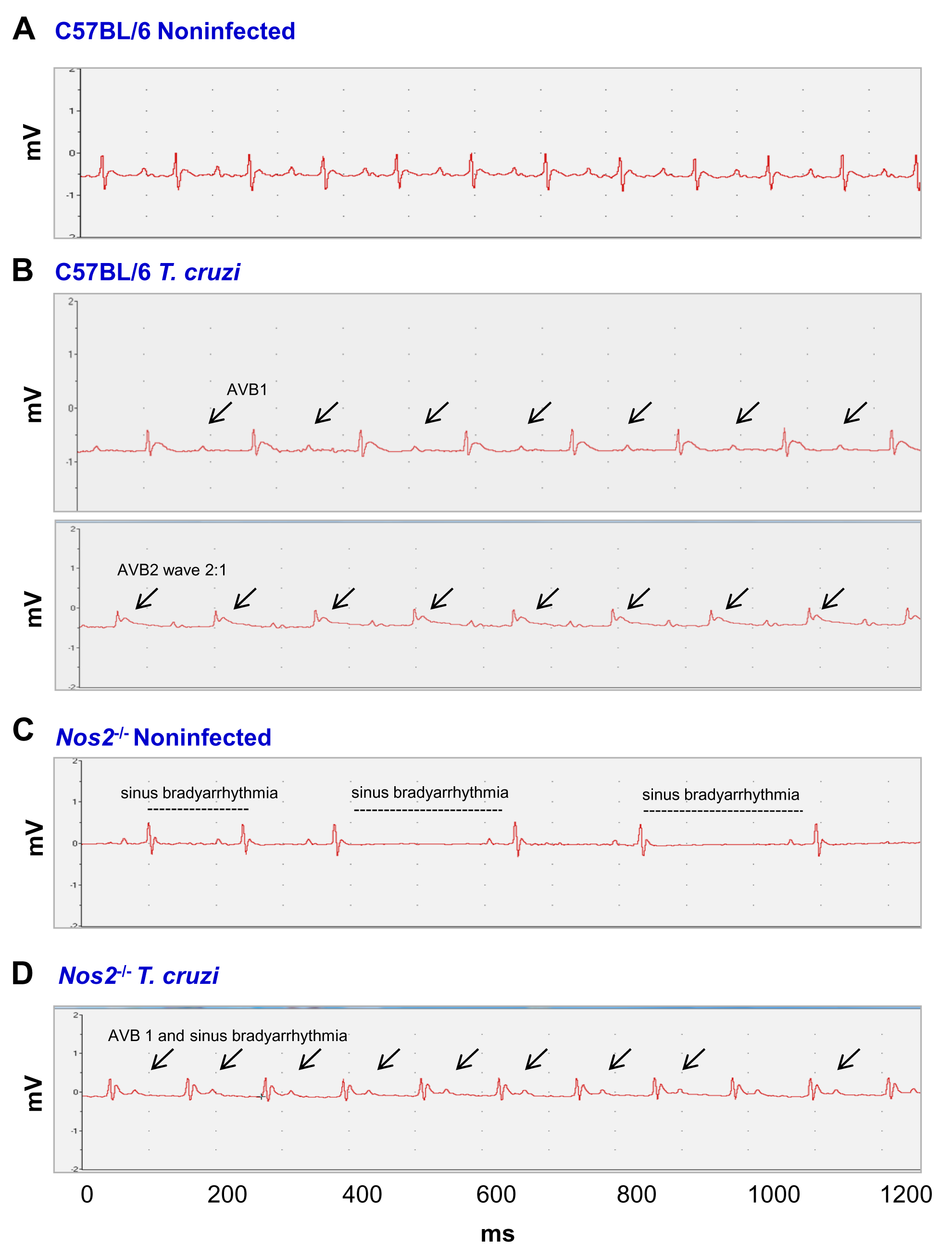

Supplement: Figure S4 — Representative electrocardiographic registers of T. cruzi -infected C57BL/6 and Nos2 −/− mice. The mice were infected with 100 blood trypomastigotes of the Colombian T. cruzi strain and analyzed at 40 dpi. (A) ECG registers of noninfected control C57BL/6 mice showing normal pattern of electrical activity. (B) ECG registers of T. cruzi-infected C57BL/6 mice showing first- and second-degree atrioventricular block (AVB1, AVB2, arrows). (C) ECG registers of noninfected control Nos2 −/− mice showing presence of sinus bradyarrhythmia. (D) ECG registers of T. cruzi-infected Nos2 −/− mice showing first degree atrioventricular block (AVB1, arrows). (TIF) [file pntd.0001644.s004.tif]
