# Supplementary material for: Inducible Nitric Oxide Synthase in Heart Tissue and Nitric Oxide in Serum of Trypanosoma cruzi-Infected Rhesus Monkeys: Association with Heart Injury
Source: PLoS Negl Trop Dis. 2012 May 8;6(5):e1644. doi: 10.1371/journal.pntd.0001644 (PMC3348164; doi:10.1371/journal.pntd.0001644)
Supplement: Table S1 — General characterization of Trypanosoma cruzi infection of rhesus monkeys. Rhesus monkeys were infected with metacyclic trypomastigote forms of the Colombian T. cruzi strain and analyzed at 20–23 years post-infection (ypi). The original colony identification used in the present study and the experimental number used in a previous publication that describes the analysis of the T. cruzi- infected monkeys during the acute infection, are provided. (DOC) [file pntd.0001644.s005.doc]

**Table S1:** General characterization of *Trypanosoma cruzi* infection of rhesus monkeys (*Macaca mulatta*)

|  |  | | | | |  | |
| --- | --- | --- | --- | --- | --- | --- | --- |
| **Experiment number** | | **Animal number in previous publication^*^** | **Animal number ^†^** | **Total inoculum Parasite**  **x 10^4‡^** | **Period post-infection** | | **Age Years** |
| I | | 3 | 42 | 1.0 | 20 | | 25 |
| II | | 5 | 64 | 1.45 | 23 | | 29 |
| II | | 7 | 68 | 1.45 | 18 | | 28 |
| III | | 9 | 90 | 1.5 | 20 | | 26 |
| III | | 11 | 95 | 1.5 | 20 | | 25 |
| III | | 12 | 99 | 1.5 | 20 | | 25 |
| III | | 13 | 103 | 1.5 | 20 | | 25 |
| Noninfected | | NI | 81 | - | - | | 25 |
| Noninfected | | NI | 94 | - | - | | 26 |

^*^ Identification according to Bonecini et al., 1990.^15^

^†^Original colony identification.

^‡^Metacyclic trypomastigote forms of the Colombian strain of *T. cruzi* were used to infect monkeys subcutaneously in the antero-lateral face of the arm.^15^
