# Supplementary material for: Inducible Nitric Oxide Synthase in Heart Tissue and Nitric Oxide in Serum of Trypanosoma cruzi-Infected Rhesus Monkeys: Association with Heart Injury
Source: PLoS Negl Trop Dis. 2012 May 8;6(5):e1644. doi: 10.1371/journal.pntd.0001644 (PMC3348164; doi:10.1371/journal.pntd.0001644)
Supplement: Table S2 — Echocardiographic patterns detected in Trypanosoma cruzi -infected rhesus monkeys during chronic infection. Rhesus monkeys were infected with metacyclic trypomastigotes of the Colombian T. cruzi strain and analyzed at 20–23 years post-infection (ypi). The echocardiographic registers were analyzed and the main findings were asynchronic interventricular septum motility and decreased left ventricular ejection fraction detected in monkey #95. (DOC) [file pntd.0001644.s006.doc]

**Table S2:** Echocardiographic patterns detected in *Trypanosoma cruzi*-infected rhesus monkeys during chronic infection.

|  | | | | | |  | |
| --- | --- | --- | --- | --- | --- | --- | --- |
| **Monkeys** | **Years p.i.** | **Heart condition** | **Years p.i.** | **Heart condition** | **Years p.i.** | | **Heart condition** |
| 42 | 18 | Normal^*^ | 20 | Sacrificed | - | | - |
| 64 | 18 | Normal | 20 | Normal | 23 | | Normal |
| 99 | 15 | Normal | 17 | Normal | 20 | | Normal |
| 103 | 15 | Normal | 17 | Normal | 20 | | Normal |
| 90 | 15 | Normal | 17 | Normal | 20 | | Sacrificed |
| 95 | 15 | AIVSM | 17 | AIVSM, ↓LVEF | 20 | | AIVSM |

^*^ECO patterns were evaluated using the following standard criteria: AIVSM - Asynchronic interventricular septum motility, ↓LVEF - Decreased left ventricular ejection fraction.
